# Measuring Attention in Rodents: Comparison of a Modified Signal Detection Task and the 5-Choice Serial Reaction Time Task

**DOI:** 10.3389/fnbeh.2015.00370

**Published:** 2016-01-14

**Authors:** Karly M. Turner, James Peak, Thomas H. J. Burne

**Affiliations:** ^1^Queensland Brain Institute, The University of QueenslandSt. Lucia, QLD, Australia; ^2^Queensland Centre for Mental Health Research, The Park Centre for Mental HealthRichlands, QLD, Australia

**Keywords:** attention, cognition, behavior, animal models, signal detection, 5CSRTT

## Abstract

Neuropsychiatric research has utilized cognitive testing in rodents to improve our understanding of cognitive deficits and for preclinical drug development. However, more sophisticated cognitive tasks have not been as widely exploited due to low throughput and the extensive training time required. We developed a modified signal detection task (SDT) based on the growing body of literature aimed at improving cognitive testing in rodents. This study directly compares performance on the modified SDT with a traditional test for measuring attention, the 5-choice serial reaction time task (5CSRTT). Adult male Sprague-Dawley rats were trained on either the 5CSRTT or the SDT. Briefly, the 5CSRTT required rodents to pay attention to a spatial array of five apertures and respond with a nose poke when an aperture was illuminated. The SDT required the rat to attend to a light panel and respond either left or right to indicate the presence of a signal. In addition, modifications were made to the reward delivery, timing, control of body positioning, and the self-initiation of trials. It was found that less training time was required for the SDT, with both sessions to criteria and daily session duration significantly reduced. Rats performed with a high level of accuracy (>87%) on both tasks, however omissions were far more frequent on the 5CSRTT. The signal duration was reduced on both tasks as a manipulation of task difficulty relevant to attention and a similar pattern of decreasing accuracy was observed on both tasks. These results demonstrate some of the advantages of the SDT over the traditional 5CSRTT as being higher throughput with reduced training time, fewer omission responses and their body position was controlled at stimulus onset. In addition, rats performing the SDT had comparable high levels of accuracy. These results highlight the differences and similarities between the 5CSRTT and a modified SDT as tools for assessing attention in preclinical animal models.

## Introduction

Cognitive symptoms are the strongest predictor of functional outcomes in patients with schizophrenia, yet current antipsychotic medications are no more effective in treating cognitive symptoms than those developed in the 1950's (Green et al., 2000; Keefe et al., 2007). To guide future clinical research in the development of more effective medications, the MATRICS (Measurement and Treatment Research for Improving Cognitive Symptoms in Schizophrenia) panel was formed (Green and Nuechterlein, 2004). For each domain of cognition, tasks administered in human subjects were selected as part of a cognitive battery for assessing the efficacy of novel medications (Nuechterlein et al., 2008). Within the domain of attention/vigilance they selected versions of the Continuous Performance Test (CPT; Nuechterlein et al., 2008). Following these recommendations the CNTRICS (Cognitive Neuroscience Treatment Research to Improve Cognitive in Schizophrenia) panel devised a similar list of tasks for evaluating these cognitive domains in animals (Carter and Barch, 2007). These tasks were selected based on evidence of face, predictive and construct validity relative to the human CPT and each has been reverse-translated back into human tasks (Demeter et al., 2008; Young et al., 2009, 2013; Worbe et al., 2014). A key issue that was raised throughout this process was the need for greater translational validity between rodent and human tasks (Hagan and Jones, 2005; Young et al., 2009). The purpose of this study was to further develop a CPT-like task for the assessment of attention in rodents.

Firstly, elements of the human CPT and dissimilarities with current rodent protocols were carefully considered. The human CPT exists in many versions with deficits in schizophrenia patients widely reported (Earle-Boyer et al., 1991; Cornblatt and Keilp, 1994). These deficits have even been suggested to represent an endophenotype of schizophrenia as there is evidence of mild deficits in first-degree relatives, stability in patients from first-episode through to remission, and a lack of correlation between severity of psychotic symptoms and CPT deficits (Chen and Faraone, 2000; Snitz et al., 2006; Gur et al., 2007; Delawalla et al., 2008; Richard et al., 2013). A common feature of continuous performance tasks is the rapid presentation of stimuli where the subject is required to monitor, identify, and respond to target stimuli. Key outcome measures are the accuracy of responding and the reaction times of participants, with both measures altered in schizophrenia patients. By translating important features of the human CPT into a rodent task, researchers can more invasively investigate how attentional deficits are related to neurobiological changes and test novel drug targets for treating cognitive symptoms in schizophrenia.

The corresponding rodent tasks selected for the domain of attention were the 5 choice serial reaction time task (5CSRTT), 5 choice continuous performance task (5C-CPT) and sustained attention task (SAT; Lustig et al., 2013). The 5CSRTT has been widely used in rats and mice with extensive investigation of the underlying neurobiology and use of pharmacological agents to probe performance (Robbins, 2002). It has been shown to be highly sensitive to pharmacological agents and to manipulations used in animal models of schizophrenia (Chudasama and Robbins, 2004; Featherstone et al., 2007; Fletcher et al., 2007; Paine and Carlezon, 2009). The 5CSRTT requires rodents to attend to an array of five apertures and make a nose poke response into an illuminated aperture. As an extension of this paradigm, the 5C-CPT incorporates a subset of trials requiring inhibition of responding when all five apertures illuminate to receive a reward. Therefore, the 5C-CPT allows the assessment of response inhibition, which is an important component of executive functioning. A clear difference between human CPT and the rodent 5CSRTT is the use of a spatial array of stimuli and response locations. In contrast, a single, constant position is typically used for presenting stimuli and responding on the CPT for human subjects. While the rodent may correctly identify the response location via a spatial stimulus-response association in the 5CSRTT, the human CPT requires the maintenance of a rule to determine the correct response based on stimulus properties. Hence, the use of a rule is a valuable feature of the rodent SAT protocol when considering translational task components. The SAT requires the detection of a single, central stimulus followed by a response on the correct lever to receive a reward (McGaughy and Sarter, 1995). On a subset of trials, a flashing light can also be presented to assess performance changes during distraction (dSAT). The SAT has incorporated the use of a rule about the properties of a single stimulus, however it has not been as widely used or validated as the 5CSRTT. In both these tasks a major issue that has not been addressed is the lack of control over the rodent's body position during stimulus presentation.

**Figure 1:**
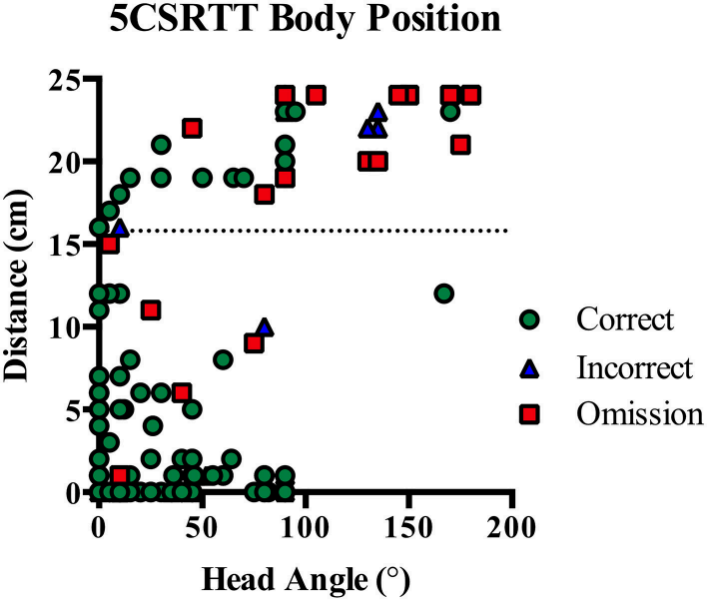
**On 5CSRTT the angle of the rat's head and distance from the stimulus are important for correct identification of the illuminated aperture**. Correct responses typically occurred when the rat was very close to or looking straight at the five-hole wall. However, rats were frequently on the other side of the chamber and looking away from the five holes prior to an omission (dotted line indicates half the width of the chamber). Plot shows individual responses from seven rats with correct, incorrect and omission responses.

In human studies the subject is often placed in a fixed position relative to the stimuli and maintains eye gaze in the direction of the stimulus stream. However, in rodent tasks, the animal can move anywhere within the operant chamber and may not have the stimuli within their visual field when it is presented. This leads to a number of differences in the interpretation of performance measures between human and rodent testing. Firstly, accuracy will depend on the body position of the rodent during stimuli presentation. However, body position cannot be determined without additional video recording and tracking analysis. Secondly, the lack of control over body position interferes with the interpretation of omission errors. In human studies an omission most likely occurs when the subject misses a stimuli due to a lapse in vigilance, whereas in rodents studies an omission may occur for any number of reasons including grooming, sleeping or investigating the chamber. To demonstrate the importance of body position, we analyzed video recordings from our previous 5CSRTT study in rats (Turner et al., 2013) and show that more omissions occurred when the rat was more distant and had their head turned during stimulus onset (see Figure 1).

Another point raised for the optimization of rodent testing included reducing the training time to encourage more widespread use (Lustig et al., 2013). Reducing the time required to train rodents on cognitive tasks would also promote preclinical screening of novel compounds, which has been limited due to the extensive investment required. Another issue that has been raised is the acknowledgment that no task provides a pure assessment of a single cognitive modality and therefore a number of behavior measures should be considered when interpreting changes in performance. Therefore, it was suggested that tasks should endeavor to include dimensions where performance can be concurrently observed over a range of difficulties, such that deficits due to more general impairments can be isolated from cognitive deficits (Lustig et al., 2013). This would serve as an internal control for changes in motivation, motoric effects of drugs and satiety as opposed to changes in attentional performance.

After considering the differences between rodent and human CPT testing and the recommendations made for optimizing task qualities, a modified signal detection task (SDT) was developed. We focussed on reducing training time, reducing omissions, and controlling body position while maintaining construct validity for measuring attention. This fast-paced SDT was designed with consideration for the species-specific differences in task performance. This includes consideration of the stimuli and response devices used, but more importantly to improve task engagement and vigilance. The SDT was directly compared to the well-validated 5CSRTT to determine the advantages and disadvantages of each paradigm. It was hypothesized that acquisition of the SDT would be faster as the task has a simpler design with fewer outcomes that are punished, thereby limiting inappropriate responding and promoting rapid task acquisition. It was predicted that there would be fewer omissions on the SDT for a number of reasons. Firstly, trials are self-initiated without delay to signal or non-signal presentation and therefore the rat should be motivated to complete each trial. Secondly, the stimuli are immediately presented directly in front of the rat and responses require only minor movement from the initial start position. Thirdly, there is minimal delay between trials. Collectively these features promote engagement in the task rather than performance of alternative behaviors. Finally, it was predicted that accuracy would be comparable on both tasks during baseline testing and when challenged with more difficult stimuli.

## Materials and methods

### Animals and housing

Adult male Sprague Dawley (ARC, WA) rats were housed in a room maintained at 21 ± 2°C and 60% humidity and on a 12-h light/dark cycle (lights on 06:00 h). They were pair-housed in polypropylene cages (41 × 28 × 24 cm) with high-top wire lids, aspen chip bedding (Able Scientific, WA, USA), nesting, and wood chew (Able Scientific, WA, USA), which was cleaned weekly after operant testing. Rats were micro-chipped (Microchips Australia Pty. Ltd., Australia) and regularly tail marked to ensure accurate identification of individuals. Prior to training, rats were food restricted to 90% of their free-feeding body weight with free access to water. Throughout testing rats were weighed daily and food rations were adjusted to maintain constant body weight. All procedures were performed with approval from The University of Queensland Animal Ethics Committee, under the guidelines of the National Health and Medical Research Council of Australia.

### Apparatus

Operant chambers were contained in sound attenuated boxes with ventilation fans (Med Associates Inc., St. Albans, VT, USA) and overhead cameras for monitoring behavior (CCD Mini CCIR, Samsung). All chambers were 50 × 50 × 50 cm and were assembled for either 5CSRTT or SDT training (Turner et al., 2013). For 5CSRTT there was a curved wall with five horizontal apertures each containing a light and head entry detector. On the opposing wall there was a house light and food magazine that was also equipped with a light and head entry detector. The arrangement for SDT training was on a single chamber wall with a house light, signal display panel and nose poke port in the middle, and a food magazine on either side of the nose poke port. The central nose poke port and magazines each contained a light and head entry detector. The signal display panel consisted of a 3 × 3 grid of light emitting diodes (5 mm, green diffuse, 80 MCD). All rats were rewarded with 45 mg grain pellets (F0021, dustless precision pellet, Bioserv, Frenchtown, NJ, USA) delivered to the food magazines. The protocols was designed using MedState Notation while operation and data acquisition was conducted using Med-PC for Windows software (Med Associates Inc., St. Albans, VT, USA).

### 5CSRTT protocol

Training for the 5CSRTT (*N* = 18) was conducted based on methods described previously (Bari et al., 2008; Turner et al., 2013) with a summary presented in Table 1. In the 5CSRTT protocol rats were required to attend to an array of five apertures and respond with a nose poke to the aperture that was briefly illuminated. Rats must withhold from responding during the inter-trial interval (ITI) where a premature response resulted in a time out (5 s). One of the five apertures was then briefly illuminated and the rat must respond within the limited hold (LH) period (5 s). Following selection of the correct aperture the rat received a food reward, however if an incorrect aperture was chosen there was a brief time out (5 s). Unlike the methods described elsewhere, prior to training stage 1, this protocol required rats to be habituated to the chambers and collect reward pellets from the apertures and magazine. To automate this process, rats were first trained to collect rewards from the magazine by placing 10 pellets in the magazine and every head entry resulted in the delivery of another pellet (Habituate). After achieving 100 pellets on 2 days, rats moved to level 0 where a response into any nose poke aperture results in a reward. These steps were used to automate the habituation procedure and ensure all rats had acquired the basic steps required for level 1. Rats were then trained progressively through levels 2–6 to collect rewards and respond to briefer stimuli until attaining level 7 with >80% accuracy and <20% omissions on a 1 s stimulus duration. For details of each level see Table 1.

**Table 1:** **Training requirements for the 5CSRTT**.

**Level**	**Trials**	**Session (min)**	**Stim Dur (s)**	**Time Out (s)**	**LH (s)**	**Reward Dur (s)**	**ITI (s)**	**Criteria**
Habituate	100	30	–	–	–	1	–	100 Responses, 2 days
0	100	30	–	5	30	10	0	>30 Correct
1	100	30	30	5	30	10	2	>30 Correct
2	100	30	20	5	20	5	2	>50 Correct
3	100	30	10	5	10	2	5	>50 Correct
4	100	30	5	5	5	2	5	>50 Cor, >80% Acc
5	100	30	2.5	5	5	2	5	>50 Cor, >80% Acc, <20% Om
6	100	30	1.25	5	5	2	5	>50 Cor, >80% Acc, <20% Om
7	100	30	1	5	5	2	5	>50 Cor, >80% Acc, <20% Om

### SDT protocol

**Figure 2:**
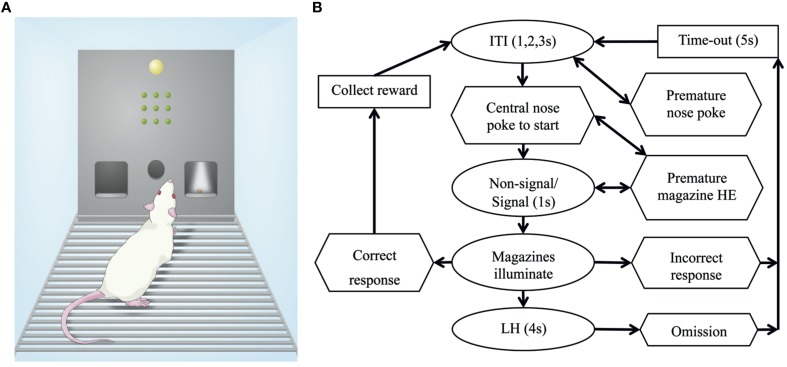
**The Signal Detection Task. (A)** Schematic of chamber arrangement including a house light, grid of lights for signal presentation, and central nose poke with a magazine on each side. **(B)** Trials started with a brief inter-trial interval (ITI) before the central nose poke aperture illuminates and the rat makes a nose poke response to begin the trial. Immediately upon nose poke detection, the signal was presented (or absent for non-signal trials) for 1 s. Following the signal presentation both the left and right magazines illuminated indicating the rat should make a choice. If the correct side was chosen a food reward was delivered, alternatively if the incorrect side was chosen there was a brief time out (5 s). If no response was made after a 4 s limited hold (LH) the trial ends with an omission scored. Nose pokes and head entries (HE) made at inappropriate times were recorded but not punished.

An overview of the SDT protocol has been presented in Figure 2. For the SDT, rats were first trained to collect a food reward from the magazines. Each subsequent head entry resulted in another reward delivery until 50 rewards were collected from each magazine or 20 min had elapsed (level 1). Next rats were trained to make a nose poke into the illuminated central port to activate reward delivery on head entry in the magazines (level 2). Finally, rats were trained to make a central nose poke, then the stimulus panel illuminated (signal trial) or remained off (non-signal trial) before both magazines illuminated and the rat could respond left or right (level 3, then level 4). A summary of the training step requirements has been listed in Table 2.

**Table 2:** **Training requirements for the SDT**.

**Steps**	**Level**	**Trials**	**Session (min)**	**Stim Dur (s)**	**Time Out (s)**	**LH (s)**	**ITI (s)**	**Criteria**
1.	Collect pellets	100	20	–	–	–	–	≥80 Trials, 2 days
2.	Nose poke	100	30	–	–	–	0,2,4,6,8,10	≥80 Trials, 2 days
3.	Signal detection training	120	30	Unlimited	–	–	1,2,3	≥80% Correct, 2 days
4.	Signal detection	120	30	1	5	4	1,2,3	≥80% Correct, 2 days

If the correct side was selected a reward was delivered; however if the incorrect side was selected a brief time out (5 s) delayed the beginning of the next trial. Stimulus (signal or non-signal) and magazine (left or right) pairings were balanced across the cohort, but constant for an individual. By incorporating the central nose poke to start trials, the rat's body position was confined to directly beneath the panel when the stimulus was presented. The location of the magazines on either side of the nose poke also reduces the amount of movement required to respond. During development of the task it was observed that if responses could be made immediately when stimuli were presented, more impulsive and inaccurate choices were made during training (data from pilot study not presented). As a result some animals did not learn the rule, preferring to respond quickly with 50% chance of success. Therefore, the inclusion of a 1 s stimulus presentation window when responses were not rewarded was critical to task acquisition. This also ensures that all animals are exposed to the same signal duration prior to responding, otherwise a faster response would reduce the amount of time the stimulus was presented. The session ended after 120 trials or 30 min and rats were required to achieve >80% accuracy with an equal number of signal and non-signal trials presented pseudorandomly.

There are a number of modifications that have been made in the SDT compared to other signal detection tasks, such as the SAT. Changes to the chamber design included the location of the reward, the use of nose pokes instead of lever press responding, the use of a central nose poke aperture and the LED light panel. In terms of protocol design, one of the most influential changes was the positioning of the rat in the center, underneath the light panel at stimulus onset. This ensured the rat was located in front of the stimulus when the signal was displayed. In addition, the ITI period occurs prior to the self-initiation nose poke, rather than after to encourage responding. Timed events and latencies are typically shorter than on other protocols to promote rapid trial pace (e.g., ITI). A mandatory pause (1 s) was incorporated during stimulus presentation, however as all other delays are minimal a rat can rapidly complete 100 trials without stopping if they chose too. The continuous nature of task performance differs from other rodent tasks where delays often lead to alternative behaviors.

### Signal duration manipulation

Following training, both tasks were adapted to include a variation in stimulus duration to increase attentional load. For both tasks there were 120 trials per session with 20 standard trials at the start and end of the session consisting of only 1 s stimulus for 5CSRTT and 0 or 1 s stimulus for SDT as per training. These trials could be used to assess the changes that occur across the length of the session. For the 5CSRTT the central block of 80 trials consisted of 0.5, 0.25, 0.12, or 0.06 s signal duration trials. On the SDT, the central block of 80 trials consisted of 60 signal trials of 0.5, 0.25, 0.12, or 0.06 s and 20 non-signal (0 s) trials. These parameters were selected to derive similar measurements from each task although all analyses were conducted separately for 5CSRTT and SDT. Inter-trial interval (ITI) was fixed for both tasks to increase stimulus onset predictability in both tasks.

### Behavioral measures

The primary outcome measure during training was the number of sessions required to reach criteria, which was >80% accuracy on 1 s signal duration for both tasks. Once they reached this stage a range of measures were used to compare performance including % accuracy, % omissions, session duration, trial rate, and response latency. Other measures could also be derived from each task but were not directly comparable due to differences in protocol requirements such as premature responses, latency to initiate trials, and reward latency. For the signal duration manipulation, % accuracy at each signal duration was calculated along with measures previously identified. The session was also split into three blocks including the start (first 20 standard trials), middle (80 reduced signal duration trials), and end (final 20 standard trials) blocks to investigate changes in performance that occur due to session length as compared to changes that occur due to altered stimulus duration.

### Statistical analysis

All data were analyzed using SPSS software package (ver. 20, SPSS Inc., IL, USA) and significance was set at *p* < 0.05. Task acquisition and baseline performance measures were compared using independent *t*-tests. Comparison of performance measures across blocks in the signal duration manipulation were analyzed by repeated measures ANOVA and followed with paired *t*-tests where appropriate. One rat was removed from signal duration analysis, as performance was unusually poor on the day of testing (<20% accuracy and 70% omissions on start block). Data are presented as mean ± S.E.M, ^*^p < 0.05.

## Results

### Task comparison

**Figure 3:**
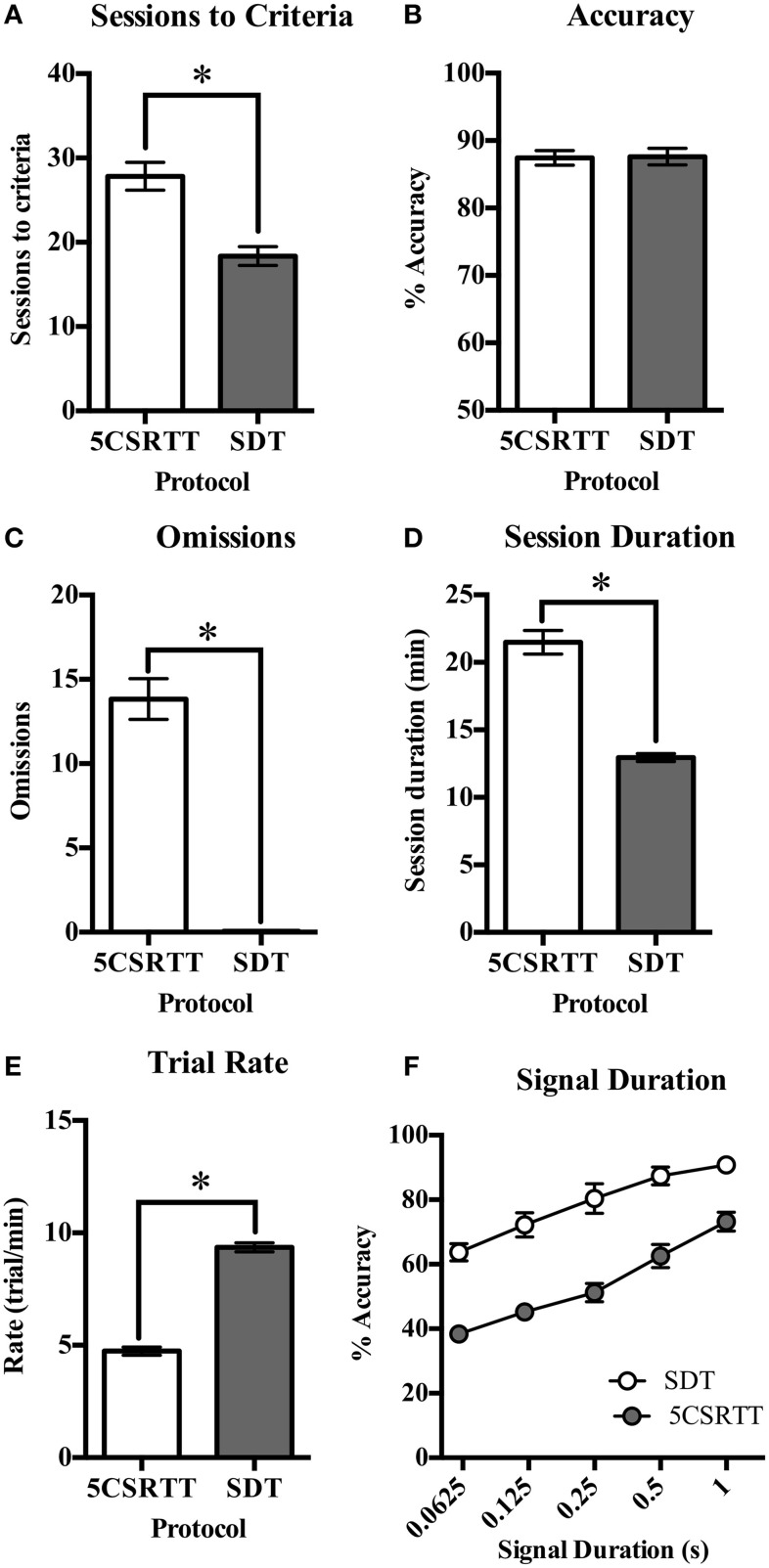
**Comparison of performance measures on 5CSRTT and SDT. (A)** The number of sessions required to train rats to the final level of 5CSRTT was significantly greater than the number of sessions required to train rats on the SDT. **(B)** Accuracy was not different between the two protocols. **(C)** The number of omissions was greatly reduced on the SDT compared to the 5CSRTT. **(D)** The average session duration was significantly longer for rats to complete 5CSRTT (100 trials) than the time taken to complete the SDT (120 trials). **(E)** This was also reflected in the trial rate, where a significantly greater number of trials were completed per minute on the SDT compared to the 5CSRTT. **(F)** The reduced signal duration manipulation led to a decrease in accuracy on both the 5CSRTT and SDT. Compared to baseline testing with only 1 s signal duration trials (Figure 3A), accuracy at 1 s remained high on SDT (from 87.6 to 90.8%) but was reduced on 5CSRTT (from mean of 87.4–73.2%) possibly due to fatigue effects (**Figure 4A**). *n* = 18/task, ^*^p < 0.05.

Performance was compared between rats trained on 5CSRTT and SDT with the number of sessions required for each training step presented in Table 3. On both tasks there were individual rats who took longer than average to reach criteria at certain steps, however to achieve an objective measure of training time on both tasks every animal was included and trained until they reached criteria. The average number of sessions required to reach criteria with a 1 s stimulus duration was significantly greater for 5CSRTT than for the SDT [*t*_(34)_ = 4.75, *p* < 0.001, Figure 3A]. Trial rate was significantly greater for the SDT than the 5CSRTT [*t*_(34)_ = −17.18, *p* < 0.001, Figure 3E] and consequently average session duration was significantly shorter on the SDT compared to the 5CSRTT [*t*_(20.72)_ = 9.17, *p* < 0.001, Figure 3D]. Both groups of rats performed to a high level of accuracy [>87%, *t*_(34)_ = −0.11, *ns*, Figure 3B], however the 5CSRTT included significantly more omissions than the SDT [*t*_(17.05)_ = 11.36, *p* < 0.001, Figure 3C]. Premature responses on the 5CSRTT were punished and therefore occurred infrequently (13.67 ± 1.36) compared with premature responses on the SDT (155.28 ± 9.75) where additional head entries were inconsequential. Non-signal trials only occur on the SDT, where accuracy was 71.9% ± 2.8, which was lower than for 1 s signal trials (90.8% ± 2.3) possibly due to greater uncertainty when perceiving the absence of a signal.

**Table 3:** **Sessions to criteria on each training step for 5CSRTT (*N* = 18) and SDT (*N* = 18)**.

**5CSRTT**				**SDT**			
**Training steps**	**Sessions, Mean ± SEM**	**Min**	**Max**	**Training steps**	**Sessions, Mean ± SEM**	**Min**	**Max**
Habituate	3.06 ± 0.21	2	5	1	3.44 ± 0.47	2	10
0	4.33 ± 0.46	2	8	2	3.39 ± 0.14	3	5
1	1.33 ± 0.16	1	3	3	8.61 ± 0.99	3	18
2	1.06 ± 0.06	1	2	4	2.89 ± 0.61	1	12
3	1.22 ± 0.13	1	3				
4	3.61 ± 0.45	1	9				
5	5.39 ± 0.76	1	13				
6	5.94 ± 0.70	2	14				
7	4.94 ± 0.86	1	13				
Total	27.83 ± 1.65	19	46		18.33 ± 1.33	12	27

### Reduced stimulus duration

**Figure 4:**
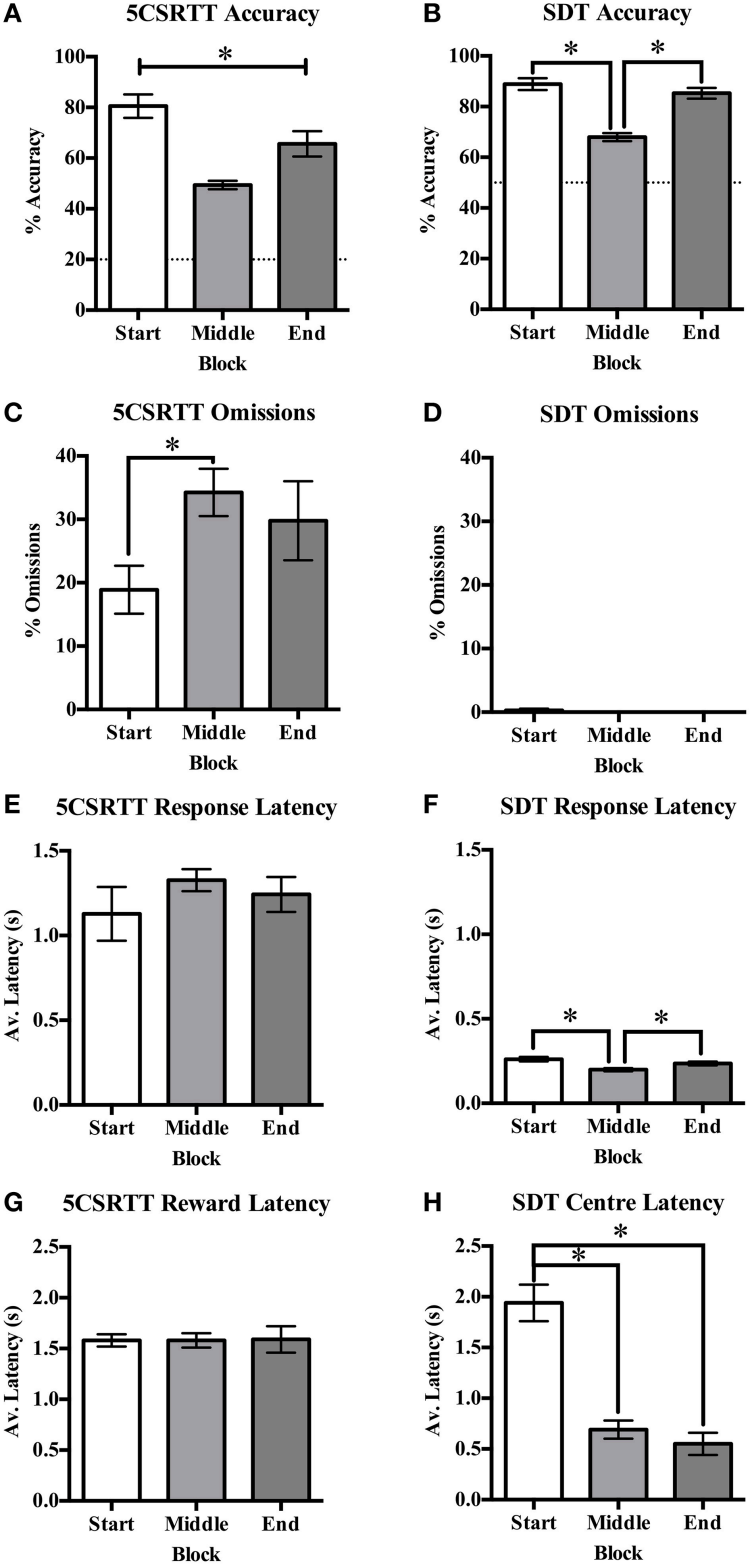
**The reduced signal duration session broken into blocks for the first 20 standard trials (start), the reduced signal durations (middle), and the final 20 standard trials (end) on the 5CSRTT and SDT**. Measures from both tasks are % accuracy **(A,B**, with dotted line indicating chance accuracy**)**, % omissions **(C,D)**, response latency **(E,F)**, and reward latency for 5CSRTT **(G)** and center latency for SDT **(H)**. ^*^p < 0.05.

**Figure 5:**
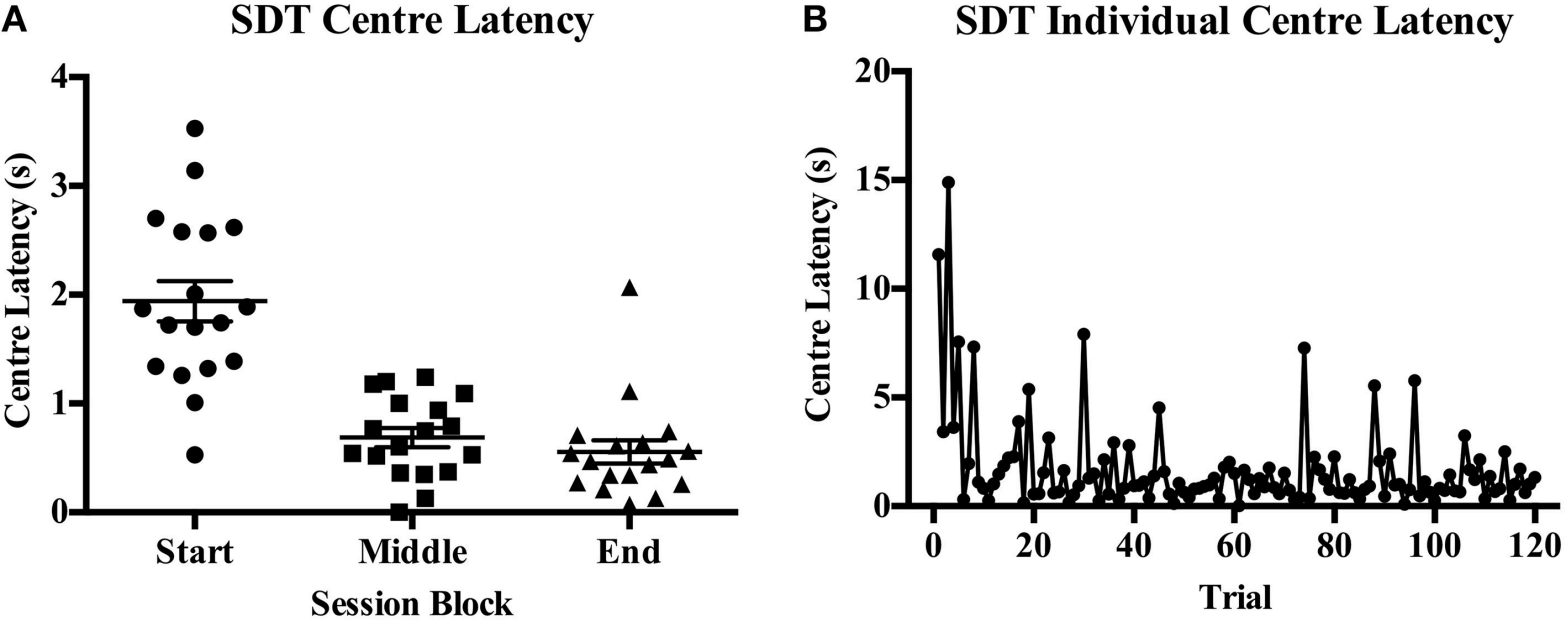
**Variability in center latency time on the SDT across session blocks. (A)** A scatterplot of the mean center latency values for each individual within each block. **(B)** A plot of an individual rat's trial-by-trial values for center latency across a session. Of interest is the occurrence of higher values in the initial trials of a session followed by more consistent, short latencies throughout the rest of the session.

Accuracy was reduced with decreasing signal duration from very high accuracy to near chance responding on both protocols (see Figure 3F for SDT). The first and last blocks of 20 trials consisted of standard trials on both tasks. On the 5CSRTT it was found that accuracy was significantly different across blocks [*F*_(2, 32)_ = 53.22, *p* < 0.001, Figure 4A] with a significant reduction from the start to end blocks [*t*_(16)_ = 4.23, *p* < 0.001], from start to middle [*t*_(16)_ = 12.16, *p* = 0.001], and from middle to end [*t*_(16)_ = −5.35, *p* < 0.001]; indicating reduced accuracy with the signal duration manipulation but also an overall decrease in performance over the session length indicative of fatigue. Omission rate varied across blocks [*F*_(2, 32)_ = 5.85, *p* = 0.07, Figure 4C], with an increase from the start to middle block [*t*_(16)_ = −4.15, *p* = 0.001], while an intermediate rate of omissions was found in the end block that did not differ from the start or middle blocks. Response latency [*F*_(2, 34)_ = 0.78, *ns*, Figure 4E] and reward latency [*F*_(2, 32)_ = 3.09, *ns*, Figure 4G] did not differ between blocks on the 5CSRTT. On the other hand, although there was a significant effect of block on accuracy on the SDT [*F*_(2, 34)_ = 37.48, *p* < 0.001, Figure 4B] this was due to the reduced stimulus duration in the middle block and was not reduced from the start to end blocks [*t*_(17)_ = 1.35, *ns*]. There was only a single rat that recorded any omissions and therefore there was no effect of block on omission rate on the SDT [*F*_(2, 34)_ = 1.00, *ns*, Figure 4D]. Also in contrast to the 5CSRTT, response latency on the SDT was reduced [*F*_(2, 34)_ = 11.00, *p* < 0.001, Figure 4F] from start to the middle block [*t*_(17)_ = 5.12, *p* = 0.001] and middle to the end block [*t*_(17)_ = −2.87, *p* = 0.011], indicating rats were responding faster across the session. In addition, center latency was altered [*F*_(2, 34)_ = 53.54, *p* < 0.001, Figure 4H] between start and middle blocks [*t*_(17)_ = 8.32, *p* < 0.001] and start and end blocks [*t*_(17)_ = 7.59, *p* < 0.001] but not middle and end blocks [*t*_(17)_ = 1.34, *ns*]. It was noted that center latency was more variable in the start block on SDT and an individual plot of center latency across trials has been provided as an example of the higher values commonly observed during the initial trials of a session (Figures 5A,B).

## Discussion

This study directly compared the performance of separate groups of rats on the modified SDT and the 5CSRTT under standard conditions and across reduced stimulus durations. Both tasks replicate features of the human CPT yet differ substantially in their protocol design. The 5CSRTT is a reaction time task with spatially separated response locations, whereas the SDT is a signal detection task where the presence or absence of a central signal indicates the correct response. In addition, a number of limitations were addressed in the development of the SDT, including reducing the time taken to train animals and limiting omissions, which may occur for different reasons in rodent tasks compared to human studies.

It was found that task acquisition to a comparable level of performance took 50% more sessions for the 5CSRTT compared to the SDT. Furthermore, the duration of each daily session on the SDT was nearly half the time taken for session completion on the 5CSRTT. Together, these findings suggest higher throughput studies would be possible with the SDT as more animals could be trained in less time. The investment of time required for operant testing is often seen as a drawback for researchers, but it is also a critical issue for preclinical testing (Young et al., 2009). Therefore, tasks that can be implemented with faster outcomes would be beneficial, as long as they are still measuring the construct of interest. Both these tasks are targeted toward measuring the construct of attention and vigilance; hence accuracy of responding was a critical outcome on the rodent tasks. Importantly, accuracy was high (>85%) and did not differ between the two tasks. On the other hand, errors of omission are more difficult to interpret in rodent studies and therefore changes were made to the SDT protocol to reduce omission rate.

To encourage responding on every trial, rats were required to initiate trials and were then immediately presented with the stimuli. This ensured the rat was positioned directly in front of the stimuli when it was presented. By contrast, on the 5CSRTT rats may be anywhere within the chamber when the stimulus is presented and inaccurate responses may occur due to poor positioning, as indicated in Figure 1. In addition, all events in the SDT occurred on the same wall of the operant chamber to reduce the amount of ambulation required, promoting rapid and continuous task performance. Overall, these protocol differences resulted in negligible levels of omissions on the SDT (<0.1%) compared to the 5CSRTT (>10%). While omission rates are an important measure on human CPT's, they are a more ambiguous outcome in rodent studies because they can occur for a number of reasons such as changes to sensory, motoric or motivational factors (Robbins, 2002). For example, rodents have been observed performing behaviors such as grooming, sleeping, and exploring while in the operant chamber. These may be considered an indicator of distractibility, but do not seem comparable with a lapse in attention as recorded by an omission in human studies. Omissions also typically increase with drug exposure, irrespective of the pharmacological target (Robbins, 2002; Paine et al., 2007). Because the rat may not be engaged in the task, it is often difficult to simply interpret the lack of response in terms of attentional processing. Other measures such as magazine head entries, trials completed as well as response and reward latencies need to be considered before suggesting that increased omissions reflect reduced vigilance (Amitai and Markou, 2010). For these reasons, it is also difficult to measure response inhibition by including withhold responses in rodent paradigms without careful task design and interpretation.

In comparing the effects of the reduced stimulus duration block, it was found that accuracy dropped as predicted for both tasks. Performance decrements can occur for a number of reasons so other variables were carefully considered to determine the likely reason for reduced accuracy. We found that the number of omissions increased when accuracy decreased during the variable signal durations on the 5CSRTT, which was also found by Fletcher et al. (2007) and has been reported in mice (Sanchez-Roige et al., 2012). Both the response latency and reward latency were unchanged in the 5CSRTT across the session indicating variable stimulus durations do not alter response speed, which is also in agreement with the literature (Fletcher et al., 2007). This indicates that the rats were not satiated or less motivated to respond across the 5CSRTT session.

By contrast, the reduced accuracy on the SDT reduced signal duration trials was not accompanied by an increase in omissions. However, both response latency and center latency were reduced during the variable stimulus duration block. Response latency was transiently reduced when stimulus duration varied. As stimuli are shorter than the standard 1 s duration, response times may be faster due to rats moving to the chosen response side at signal offset. Center nose poke latency was also reduced from the start block to the variable stimulus duration block and remained low for the final block of trials, indicating an effect of session rather than a transient shift due to changes in stimulus properties. Because occasional large latency values were seen in the start block for individual rats (see Figure 4B), we suggest this reduction in center latency time maybe due to habituation to the chamber during the first block of trials. Distractions, such as odors from the previous animal, and competing behaviors may reduce within a few trials as the rat becomes more focussed on performing the task. Despite the SDT trial rate being self-paced by the rat, the rate of stimuli presentation was roughly double that of the 5CSRTT (average inter-stimulus interval of 6.4 s on SDT vs. 12.7 s on 5CSRTT) and was more similar to the fast rate used in the human CPT (commonly ranging between 0.5 and 2 s; Riccio et al., 2002). This indicates rats are initiating and responding on trials consistently and rapidly. At face level, this type of rapid and continuous responding reflects the monotonous pattern of responding required on the human CPT.

Compared to versions of the human CPT, there are still a number of missing features. There was no response inhibition or no-go component incorporated into this task, such as that in the rodent 5C-CPT and many human CPT's. However, some versions do require responses to both target and non-target stimuli, such as the CPT in the commercially available Cogtest battery. The focus of this study was on measuring attention and given the issues associated with correctly identifying an inhibited response in rodents, this component was not included. In human CPT studies a large range of visual stimuli (e.g., the alphabet) can be used simultaneously and easily identified by subjects, whereas this is not feasible in rodents. In addition, there are many versions of CPT to tax different processes, such as working memory and cognitive control. Given the heterogeneity in human CPT design, incorporating core features like the continuous and rapid response to stimuli were the priorities in modifying the SDT. Additional modifications could be made in the future to measure other cognitive processes assessed by versions of the CPT.

Few studies have directly compared alternative paradigms to the 5CSRTT, however a recent study by Leite-Almeida et al. (2013) showed that impulsive responding on their novel Variable Delay-to-Signal task correlated with impulsivity during early stages of 5CSRTT training (although not when attentional load increased). It is important to note the 5CSRTT is very useful for measuring impulsive behavior but this has not been a priority in developing the SDT. Although inappropriate head entries can be made on the SDT, they were not punished and therefore interpretation about this behavior is quite different to premature responding on the 5CSRTT. In addition, preservative responding was easily measured with the 5CSRTT however this was not possible with the SDT as response and reward occur together. Therefore, if impulsive or compulsive behaviors are of interest, the 5CSRTT should be used. With the recent adaptation of many rodent tasks to use touchscreens, more tasks may be directly compared to the 5CSRTT using a battery approach (Hvoslef-Eide et al., 2015). Unfortunately, as the touchscreen chambers utilize an entire wall for stimulus display, rewards are delivered on the opposite side of the chamber to stimulus presentation. This limits the inclusion of modifications made in this study to control body position and promote fixation.

Another rodent task designed to measure attention is the sustained attention task (SAT or dSAT when a distractor is included; McGaughy and Sarter, 1995). This task is also a type of SDT, however there are a number of differences. In construction, these include the position of the reward magazine relative to the response panel and the use of levers in the SAT rather than nose poke receptacles in the SDT (McGaughy and Sarter, 1995). By providing the reward on the same side of the chamber, ambulation was reduced allowing the rat to remain in front of the stimulus throughout training. This allows sessions to run at an increased task pace and promotes vigilance through stillness, as in human CPT testing. More recent SAT papers indicate the reward delivery system has been moved to the same wall as stimulus presentation (Demeter et al., 2008; Paolone et al., 2013); however the reward is provided in a central position rather than in the location of the correct response and there is not a separate port for trial initiation like in the SDT. Additionally, on the SDT the stimulus was presented when the rat makes a central nose poke directly underneath the light panel, controlling body position within the chamber. By comparison, stimulus onset on the SAT occurs after a variable ITI during which time the rat can move anywhere within the chamber. The issue of body orientation has been acknowledged as ITI was reduced (from 12 ± 3 to 9 ± 3 s) and stimulus duration was reduced (from 1 to 0.025–0.5 s) in an attempt to “constrain their behavior and presumably maintain persistent orientation toward the intelligence panel” (Demeter et al. (2008), p. 790). The self-initiation of trials also promotes trial completion and as an example omission rate on SAT has been reported around 2.5%, whereas on SDT it was 0.05% (Demeter et al., 2008). With the administration of pharmacological agents that typically increase omissions, self-paced trials on the SDT will allow the separation of inability to complete a trial from motivation or ability to start trials. The inclusion of levers has also limited the use of the SAT in mice, where a nose poke receptacle may be favorable (St. Peters et al., 2011). We have successful trained and tested pharmacological agents in two mouse strains using the SDT protocol with only minor changes, such as reward type, as the equipment used and protocol parameters were originally selected to accommodate both species (pilot study, unpublished). Other differences include the time schedule used. On the SDT, rapid trial rate was promoted through limited ITI's of 2 ± 1 vs. 9 ± 3 s on the SAT. Substantial training is required for stable levels of performance on the SAT, with the suggestion that 4–8 months was required when training 5–6 days per week. This is around 80-200 sessions and significantly longer than the SDT training reported here, albeit stability criteria on each task have not been matched (Arnold et al., 2003). Therefore, there are a number of differences between these three tasks measuring attention that create unique forms of rodent performance and outcomes.

## Conclusions

In summary, compared to the 5CSRTT, there were fewer training sessions and reduced session duration on the SDT, allowing higher throughput testing of animals. In preclinical settings this would reduce the time taken to test compounds, while in a research environment with limited operant chambers this would allow larger cohorts to be tested. Omissions can be difficult to interpret, particularly when they typically increase after drug administration, and hence modifications were made to reduce omissions on the SDT. We have also demonstrated that manipulating the signal duration leads to a comparable reduction in accuracy across both tasks. Importantly, we have controlled body position in relation to stimulus presentation and encouraged rapid trial progression on the SDT to emulate features of the human CPT. This study highlights key differences and similarities between the traditional 5CSRTT and a SDT modified to meet modern demands.

## Author contributions

KT, JP, and TB designed the study. JP and KT conducted the experiments. KT and TB analyzed the data. KT, JP, and TB contributed to manuscript production.

## Funding

This work was supported by the National Health and Medical Research Council of Australia. KT was supported by the Queensland Government Smart Futures Ph.D. Scholarship and an Australian Postgraduate Award.

### Conflict of interest statement

The authors declare that the research was conducted in the absence of any commercial or financial relationships that could be construed as a potential conflict of interest.
